# Does a Dog at School Help Identify Human and Animal Facial Expressions? A Preliminary Longitudinal Study

**DOI:** 10.3390/ejihpe15020013

**Published:** 2025-01-30

**Authors:** Manon Toutain, Nicolas Dollion, Laurence Henry, Marine Grandgeorge

**Affiliations:** 1 EthoS (Éthologie Animale et Humaine)—UMR 6552, Centre National de la Recherche Scientifique (CNRS), University Rennes, Normandie University, F-35000 Rennes, France; laurence.henry@univ-rennes.fr (L.H.); marine.grandgeorge@univ-rennes.fr (M.G.); 2 Laboratoire C2S (Cognition Santé Société)—EA6291, Université Reims Champagne-Ardenne, F-51100 Reims, France; nicolas.dollion@univ-reims.fr

**Keywords:** facial expression, identification, school, adolescent, animal, cognitive disorders, service dog

## Abstract

(1) Background: Animals provide many benefits in children’s lives, but few studies assess the effects of animal presence—especially service dogs—in schools. This pilot study examined whether a year-long exposure to a service dog could improve facial expression recognition in adolescents with cognitive function disorders. (2) Method: Twenty-three adolescents participated: 10 with cognitive function disorders who were part of a specialized French teaching program (LUSI) that included a service dog (LUSI group), and 13 neurotypical adolescents who served as controls (not in LUSI, no service dog exposure). Participants assigned one of five facial expressions (sadness, joy, fear, neutral, anger) to images of human, dog, and cat faces at three intervals: before dog integration, at 5–8 months, and 11–14 months later (same intervals for controls). (3) Results: Identification of facial expressions of both dog (*p* = 0.001) and human (*p* = 0.01) but not cat (*p* > 0.05) faces by LUSI participants exposed to service dog improved with time. The performance of LUSI participants was better when they lived with various species of animals at home. Control participants’ performance did not change significantly (all *p* > 0.05). (4) Conclusions: After a school year, the presence of a service dog had helped adolescents with cognitive function disorders to better identify human and dog facial expressions.

## 1. Introduction

The ability of humans to perceive and understand the mental states of others has been a decisive evolutionary mechanism facilitating social interactions, thus optimizing individual survival, notably through cooperation (Mafessoni & Lachmann, 2019). Understanding others’ mental states is closely intertwined with recognizing and responding to their emotions, as both are fundamental to navigate in social situations.

Vision is the primary modality used by humans to recognize others’ emotions (Ferretti & Papaleo, 2019; Russell & Fernández-Dols, 1997) and faces convey a great deal of information that can communicate a wide range of emotions that others can perceive and interpret. Authors have evidenced that some basic facial expressions are universal (i.e., anger, disgust, fear, joy, sadness, surprise; Cowie et al., 2001; Ekman, 1994; El Ayadi et al., 2011). Thus, facial expressions are a distinct and specific form of signal among the various ways emotions can be conveyed. A multitude of facial muscle movements (with variations among individuals, but with conformational characteristics in common) revealing emotional expressions can be interpreted by others as emotional signals (Dimberg et al., 2000; Ekman, 1993). The universality of these emotional facial expressions suggests that their recognition could be innate, but this is not the case: facial emotion recognition is the result of the gradual development of an expertise that young children acquire through experience (Kolb et al., 1992). This development begins early in life and this skill continues to develop throughout childhood and adolescence, with improvements occurring in parallel to the maturation of the frontal cortex (Kolb et al., 1992; Malsert et al., 2020).

### 1.1. Children’s and Adolescents’ Skills to Recognize Pets’ Facial Expressions and Emotions

Processing and understanding emotional facial expressions do not apply solely to intra-specific interactions. They are also present during interactions with non-human partners. In western countries, animals are part of our daily lives and one household in two owns a pet, the most common worldwide being dogs and cats (i.e., 43% families have no pet, 33% have dogs, and 23% have other pets) (Growth from Knowledge, 2016). Thus, children and teenagers often have opportunities to interact with both human and non-human partners. Just like humans, animals emit various communication signals, mainly non-verbal via facial expressions (e.g., pain: rabbit (Keating et al., 2012), horse (Costa et al., 2014), sheep (McLennan et al., 2016), rat (Sotocinal et al., 2011)).

Adults can recognize dogs’ basic emotions, particularly fear, expressed on their faces and by their eyes, but identification of cats’ facial expressions varies, adult women performing better (Bloom et al., 2021; Dawson et al., 2019). Young children (i.e., 3–6 years old) may have difficulty interpreting a dog’s behaviour, and this can induce higher risks of being bitten (Meints et al., 2010, 2018). Amici et al. (2019) mentioned that 5- and 6-year-old children are able to recognize certain canine emotional facial expressions such as anger (Amici et al., 2019). Nevertheless, these abilities remain limited regardless of the subjects’ general experience with dogs. These results echo another study when 4- to 10-year-old children were asked to recognize five facial expressions (joy, positive anticipation, fear, frustration, and neutral) on videos of human and dog faces (Correia-Caeiro et al., 2022). Six- to 12-year-old children evaluated audio recordings of dogs (recorded while the dog was placed in specific situations triggering distinct emotional states) approximately 60% more accurately, and audiovisual recordings 45% more accurately, than did 4- to 5-year-old children (Eretová et al., 2020). Overall, authors report that children identified humans’ emotions better than dogs’ emotions, but that with development and experience their skills develop (Eretová et al., 2020; Correia-Caeiro et al., 2022; Törnqvist et al., 2023; Meints et al., 2010). To our knowledge, no study has yet focused on children’s recognition of cat emotions.

As for human facial expressions, multiple variables—such as age, gender, race, experience—can influence the processing of animal facial expressions. Törnqvist et al. (2023) showed that the ability to evaluate dogs’ emotions varied with participants’ age. Adults already familiar with dogs recognized canine emotional expressions more successfully than 4-year-old children with experience of dogs, but no better than experienced 6-year-old subjects. Similarly, Aldridge and Rose (2019) observed that 4- to 5-year-old children performed less accurately than their 6- to 7-year-old counterparts (Aldridge & Rose, 2019). Reports showed that younger children, aged 4 to 6 years, have difficulty identifying fear in dog pictures and videos, whereas 8 to 10-year-old children performed better, although their performance was not on par with that of adults (Lakestani et al., 2014). Altogether, these studies showed that children’s ability to recognize canine emotions and their strategies evolved with age, with a notable improvement between the ages of 4 and 10; their performance generally remained inferior to that of adults. Like age, life experience can have an influence. Skill to recognize dogs’ emotions is influenced by experience with them and seems to progress with experience, as well as to be linked to the progressive development of the brain structures responsible for facial emotion recognition. Nevertheless, although the interpretation of signals emitted by dogs or cats can vary according to individual’s experience with these animals, owning a dog or a cat does not seem to predict better identification of their signals. This has been observed in studies involving adults, such studies using videos of dog behaviours (Tami & Gallagher, 2009; Wan et al., 2012), and videos of cat facial expressions (Dawson et al., 2019). Gender differences exist in the processing of human emotional facial expressions; women often recognize non-verbal emotions better (Herba & Phillips, 2004; McClure, 2000). However, analyses of recognition of canine emotions on videos suggest that gaze patterns do not differ according to children’s gender (Correia-Caeiro et al., 2022). These findings suggest that while gender may influence humans’ recognition of emotions, its impact on interpreting animal emotions appears less pronounced. Dogs present a wide variety of physical characteristics and morphologies (Wayne & vonHoldt, 2012) and their faces vary considerably from one breed to another. This is not just limited to differences in size, shape, and skull structure, but encompasses aspects such as colour, length, and type of hair covering the face. As a result, facial expressions and the features it involves can change in relation to a breed’s physical characteristics (Correia-Caeiro et al., 2022). Breed had an influence on adults’ identification of dog facial expressions (Bloom et al., 2021; Burza et al., 2022). A less studied factor is the influence of an individual’s developmental status, e.g., the presence of cognitive or developmental disorders (e.g., McAlpine et al., 1991; Mcalpine et al., 1992; Rojahn et al., 1995). For example, Autism Spectrum Disorder (ASD) is a neurodevelopmental disorder specifically characterized by deficits in social interaction and communication, which notably include difficulty identifying and understanding other humans’ emotions (DSM-5; American Psychiatric Association, 2013; Celani et al., 1999; Harms et al., 2010; Kuusikko et al., 2009). Interestingly, use of animal filters improved the ability of 12- to 17-year-old adolescents with ASD to identify emotions on human faces (Cross et al., 2019). In the same way, individuals with attention deficit hyperactivity disorder (i.e., ADHD) made mistakes when interpreting facial movements, tone of voice, and gestures conveying either positive or negative emotions (Lievore et al., 2023; Ludlow et al., 2014; Pelc et al., 2006). Some individuals with disabilities, for example intellectual development disorders and conduct disorders, frequently encounter more challenges when trying to discern emotions on facial expressions than do subjects without disabilities (e.g., McAlpine et al., 1991; Mcalpine et al., 1992; Rojahn et al., 1995). However, to our knowledge, nothing is known about their abilities to identify animal facial expressions.

### 1.2. The Benefits of Animals for Young People with Cognitive Disorders and the Specificity of Face Processing/Animal Expressions

The presence of animals in children’s daily life brings many benefits during their development (Ávila-Álvarez et al., 2022; Purewal et al., 2017; Dollion & Grandgeorge, 2022). In particular, repeated or sporadic interactions with animals offer significant benefits to children with Neurodevelopmental Disorders (NDD) by promoting their social, emotional and cognitive development (e.g., ASD, Carlisle, 2015; Lisk et al., 2021; Dollion & Grandgeorge, 2022). Children with ASD show a particular visual attraction to dog faces suggesting an interesting potential to use interactions with this species in interventions with these children. In the same way, a case study of two children with ADHD accompanied, for one year, by a dog in the classroom through an animal assisted education program showed marked improvement of their attentiveness (Juríčková et al., 2020).

Altogether these results raise the question of whether the presence of and daily interactions with animals influence the skills of subjects with atypical development or cognitive disabilities to identify emotional facial expressions. Therefore, we analysed the influence of the presence of a service dog in the daily school life of adolescents with atypical development on their skills to identify the facial expressions of different species. The choice of the school context and the service dog was based on previous studies. More and more schools integrate animals, and reports show that this integration has numerous benefits, especially for atypical students (Brelsford et al., 2017). A decrease of students’ anxiety in the presence of an animal in the classroom has been observed (Herbert & Lynch, 2017) along with increase of their academic motivation (Beetz, 2013) and reduction of their aggressive and disruptive behaviours (Hergovich et al., 2002; Kotrschal & Ortbauer, 2003). Many studies of the effects of an animal in a classroom are based on teachers’ perceptions via questionnaires, with very few direct tests including students (Gee et al., 2017; Simard & Deneault, 2022). In addition, the protocols are very heterogeneous and no study has included longitudinal assessments using direct tests of skills (Brelsford et al., 2017). Moreover, no study focused primarily on the effects of animals on students’ ability to identify emotions or facial expressions. Thus, our longitudinal study compared 10 adolescents with disabilities included in a Localized Units for School Inclusion (LUSI) program (i.e., a specialized teaching program) with a service dog and 13 adolescents without disabilities not included in this program (control group), all tested using a computerized facial expression identification task at three different times during the school year. We predicted that the skills of the LUSI group, exposed daily at school to a service dog, to process the facial expressions on dog and human faces would improve over the year, whereas the skills of the control group would remain stable. This would translate into improved accuracy and decreased variability in confusion regarding facial expressions. Exposure and interaction with the service dog on a daily basis should enable the LUSI students to hone their identification of facial expressions. Similarly, it should reduce the variability of the types of misidentifications, as repeated interactions with the service dog would enable them to become more familiar with canine facial expressions. Due to their social catalyst effect, dogs induce more interactions between humans, which in turn should lead to improvements in recognition of human facial expressions (McNicholas & Collis, 2000; Dollion & Grandgeorge, 2022). We argue that these abilities would extend to other species, which is why we also tested identification of cat facial expressions.

## 2. Materials and Methods

### 2.1. Ethics

The present research was non-invasive and did not involve pharmacological interventions. Therefore, in line with the recommendations of the Ethics Committee, parents provided their informed written consent. Adolescents’ oral and written assent was collected as well. This study was declared in compliance with the MR-004 methodology of French law: A declaration to the DPO (Déléguée à la Protection des Données) was made on 30 March 2022, the CNIL declaration was declared compliant on 23 May 2022, and a file was submitted to the HDH (Health Data Hub) on 25 May 2022. Finally, this study was validated by the Sud Mediterranéen III ethics committee on 5 September 2022, [N°2022.06.09 bis_22.02106.000105].

### 2.2. Participants

Observations and data collection were performed from May 2022 to June 2023.

#### 2.2.1. Adolescents

Twenty-three adolescents (10 girls and 13 boys), all in the same junior high school in France, between 11 and 15 years old (M = 12.9, SD = 1.2 years-old) participated in this experiment (see details, Table 1).

To be included, they had to pass a visual acuity test; all adolescents had normal or corrected-to-normal eyesight. Ten were adolescents with cognitive disorders, benefiting from a special teaching Localized Units for School Inclusion (LUSI) program in France (M = 13.3, SD = 1.5) and regularly exposed to an Assistance Dog for School Success (i.e., service dog) (LUSI group) (Table 1a). Thirteen additional adolescents, neither included in the LUSI program nor exposed to a service dog (control group) (M = 12.5, SD = 0.8), took part in this study (Table 1b). No significant difference was observed between the mean ages of the two groups (Mann–Whitney test, W = 84, *p* = 0.233). Information relative to adolescents’ visual attention and the presence of animals at home was collected through a questionnaire completed by parents. Parents also completed the French version of SCQ (Social Communication Questionnaire; French version: Kruck et al., 2013; initial version: Rutter et al., 2003) and the Dunn sensory profile (short version for ages 3–10 years and 11 months) (Dunn, 2010). The number of species present in the household did not differ significantly between the two groups (W = 72.5, *p* = 0.9068). The control group included eight adolescents with cat(s), six with fish(es), two with chicken(s), six with dog(s), one with rabbit(s), one with hamster(s), and one with guinea pig(s). The LUSI group included seven adolescents with cat(s), two with fish(es), one with horse(s), three with dog(s), and three with rabbit(s).

#### 2.2.2. Service Dog

The service dog was a male golden retriever born on 19 August 2020 and nearly two years old when integrated in the LUSI program. He had received specific training from the French association Handi’Chiens to become a service dog for academic success (i.e., 18 month of training). This service dog joined the LUSI program in May 2022. The LUSI teacher, who was the prime referent for the service dog, was trained by the association to provide the commands to which the dog responded and to handle the general care of the dog. The service dog was present in the classroom during the same hours as the teacher (i.e., 21 h/week). Adolescents in the LUSI program could request its presence at any time. In the LUSI program, the service dog alternated between active and passive presence. During active presence, the service dog participated in educational activities led by the LUSI teacher, such as reading aloud to the service dog, illustration of concepts using or interacting with the service dog, or routine-like activities with the service dog (e.g., removing the harness, providing water) and play activities with the service dog. During passive presence, the service dog moved freely in the classroom, engaging in spontaneous interactions (e.g., petting sequences or spontaneous play), or resting in its basket (a necessary resting period was respected to ensure the service dog’s well-being), always remaining visible to the adolescents. During outings, a ritual involving the harnessing of the service dog and the use of a double leash (one held by the teacher and the other by the adolescent) ensured safety while engaging the LUSI adolescents. In contrast, adolescents in the control group encountered the service dog only during two specific situations: either during the test sessions held in the LUSI classroom, or during quick encounters in the hallways. In these cases, interaction or direct contact occurred between the control group adolescents and the service dog.

In the LUSI program, zoonotic risks were minimized through regular veterinary check-ups, up-to-date vaccinations, and parasite prevention. The welfare of the service dog was ensured through constant access to water, a resting area, and the possibility to freely withdraw from interactions when needed. The service dog benefited from 1.5 h of daily walks, divided into three to four outings, and returned to the teacher’s home every evening. The LUSI teacher had received a one-week training from the Handi’Chiens Association to recognize the service dog’s needs and signs of stress, with annual refresher sessions to maintain best practices. Additionally, the service dog’s long-term well-being was monitored through annual questionnaires sent by the Handi’Chiens Association and completed by the LUSI teacher and a veterinarian.

### 2.3. Visual Stimuli

**Figure 1:**
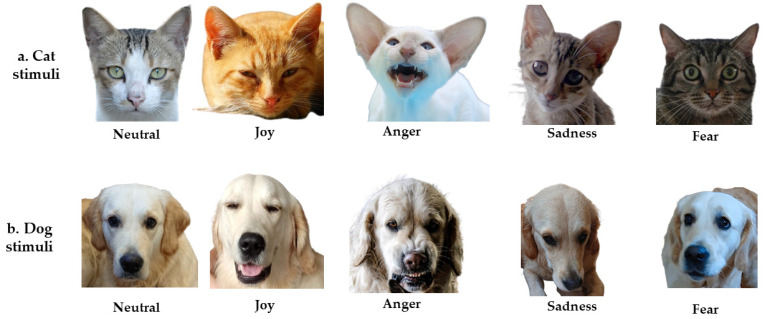
(**a**) Example of cat stimuli (sources: Neutral—Thinkstock/Getty Images (modified) and Borgi et al. (2014); Anger—Pxhere (modified, background removed); Joy, Fear, and Sadness—Kaggle (modified, background removed). (**b**) Example of dog stimuli, specifically featuring the Golden Retriever breed (Anger—Inès Sauvage; other emotions—personal sources). Illustration of human stimuli was not permitted, as they were sourced from the FACES data bank.

Three categories of photographs were used: dog faces, human faces, and cat faces. The photographs of dogs included five pictures of the same golden retriever (i.e., same breed as the service dog) depicting five different emotions (i.e., sadness, joy, fear, neutral, anger) and five pictures of different dogs of different breeds, each depicting a different emotion. The photographs of cats included 10 pictures of different breeds, depicting five different emotions (Figure 1a,b as example). Finally, the photographs of humans represented five women and five men, also depicting five different emotions. The photographs of dogs and cats were sourced from Bloom and Friedman (2013); Borgi et al. (2014), Inès Sauvage, Pxhere, Kaggle, and authors’ personal sources. The photographs of humans were extracted from the FACES databases. Thus, our complete test set included 30 photographs of three species, depicting five different facial expressions (i.e., sadness, joy, fear, neutral, anger) with two exemplars of each emotion. Disgust and surprise (two so-called “universal emotions”; Ekman, 1994) were not included, as dogs’ and cats’ facial expressions of disgust are difficult to provoke, whereas surprise is similar to and easily confused with fear. Moreover, according to Taylor et al. (2015) individuals with developmental impairments, such as ASD and specific language impairments, have more difficulties recognizing surprise and disgust.

The background was removed from all dog and cat photographs and all the human photographs had a grey background. All photographs showed individuals front facing. Pictures sizes varied: the dog photographs were 10.3 ± 1 × 9.9 ± 0.67 cm, the cat photographs were 10.7 ± 0.8 × 11.3 ± SD0.9 cm and the human photographs were 19 × 15.2 cm. The photographs were selected after presentation to six adults who reached an inter-observer rating agreement to ensure correct emotion identification.

### 2.4. Procedure

All experimentations were carried out at the participants’ school, always in the same classroom (i.e., the LUSI class). The experimenter (MT) first asked the adolescents if they agreed to perform the test. Once consent was obtained, each adolescent was seated on a chair facing a table with a computer always placed in the same position within the LUSI classroom setup. The room had closed curtains and was lit by artificial lighting. The experimenter (MT), also always sat in the same position, to the adolescent’s right, to provide support during the test. The visual stimuli were presented on a 13-inch computer screen (Dell Latitude 5420 computer). Before starting, participants were asked to tell the experimenter their definition of each of the five facial expressions (i.e., sadness, joy, fear, neutral, anger). Then, the experimenter (MT) gave a definition of the facial expressions without referring to any facial feature involved. Once the participant was placed in front of the computer, the experimenter followed a standardized script and stated: “I’m now going to show you a series of photos of faces. For each face, you’ll have to tell me what emotion you think the face is expressing. To help you, I have put in front of you the names of the possible facial expressions”. Rectangular labels showing the five facial expressions were placed in front of the participant (i.e., individualized plasticized rectangles with names of one the five facial expressions written on it). These rectangles were arranged horizontally from left to right, rearranged for different participants and replaced randomly between trial categories (but not within each stimulus category). The series of stimuli were presented in blocks. Selection of block order was randomized (e.g., participant starts at (t1) with all the cat, then dog, then human stimuli; at (t2) the order changed and could be dog, human, or cat stimuli). Within each species block, the order of facial expressions was randomized across participants and sessions. A participant’s response could be either verbal or non-verbal (i.e., pointing to the label). Participants were motivated to complete the task and, in retribution for their participation, received a sticker. The participants were under no time pressure to respond. The experimenter presented the next stimuli after the participant had answered. The entire testing session was completed within a maximum of 10 min. Participants’ responses were recorded on paper by the experimenter. Participants’ answers were then transposed into a binary score: 1 = success, 0 = failure.

Each participant of the LUSI and control group completed the task at three measurement times:

- Baseline, (t1) = before the service dog was integrated into the LUSI program (for six participants of LUSI group and for all participants of control group) or before establishment of a relationship with the service dog (less than 10 days of exposure to the service dog, for four participants who integrated the LUSI program later during the school year).
- Midpoint, (t2) = 5–8 months after integration of the service dog
- End point, (t3) = 11–14 months after integration of the service dog

All participants from the control group completed the task during the same periods as the LUSI group.

### 2.5. Statistical Analyses

Statistical analyses were performed using RStudio software version 2023.09.0. The significance threshold was set at *p* < 0.05 for all analyses. Analyses were conducted following three steps.

Variations of identification accuracy according to group, species, and facial expression at (t1) and (t3) were estimated using a GLMERbinomial Model (i.e., generalized linear mixed-effects model with binomial logistic regression), built using the GLMER function in R (“lme4” package). The variable to be explained was the binary right/wrong answer variable, the fixed effects were: group (LUSI/control), species (dog, cat, human) and type of facial expression (joy, neutral, fear, anger, sadness). Participants were included as random factors. A type II ANOVA (“Anova” function in R (“car” package)) was applied on the model to test the significance of the fixed factors. Tukey tests were applied as a post hoc analysis using the “glht” function in R (“multcomp” package).

As the first step of the analysis revealed an effect of the group variable (see results section) at (t1) but not at (t3), distinct GLMERbinomial Models were run, but on each group this time (i.e., LUSI and control). The variable to be explained was the binary right/wrong answer variable, the fixed effects were: session (t1, t2, t3), species (dog, cat, human), and facial expression (joy, neutral, fear, anger, sadness). Participants were again included as a random factor. A type II ANOVA was applied on the model to test for the significance of the fixed factors. To assess differences between the levels of a variable (e.g., facial expression, species) in the model, post hoc analyses (“glht” function in R, “multcomp” package) were carried out using the Tukey method.

To describe how the accuracy for each emotion varied within each group and across the three species, and to evidence differences between species for each facial expression McNemar’s Chi-squared tests were performed.

GLMERbinomial Models were applied within each group and each species to evaluate the temporal evolution of participants’ accuracy and to investigate the possible influence of other factors. The variable to be explained for each condition (i.e., each species and group) was the binary right/wrong answer; the fixed effects were age, sex, session, facial expression, the number of different species owned by the participant, SCQ score, and “breed” was included for the dog condition. Participants were included as random factors. Distinct models were computed for each species and each group. The variance inflation factor (VIF) was measured following the mixed models to assess potential multicollinearity between variables. At all steps, a type II ANOVA was used to test the significance of the fixed factors. To assess differences between the levels of a variable (e.g., facial expression, session) in the model, we computed a post hoc analysis using the “glht” function in R using the Tukey method. Parallel to the accuracy scores in this third step, Chi-squared tests of independence were applied to characterize the type of errors made for each species and to compare the types of errors committed within each group at (t1) and (t3).

## 3. Results

### 3.1. Accuracy at (t1) and (t3): Differences Between Groups, According to Species and Facial Expression

#### 3.1.1. Baseline at (t1)

The GLMER_binomial_ Accuracy ~ Group + Species + FacialExpression + (1|Individuals) model was used. This model showed that during the first session (t1), **group**, **species**, and **type of facial expression** influenced facial expression identification accuracy. The GLMER model showed a significant difference between the LUSI group and the control group. The LUSI group participants had lower accuracy scores than the control group participants (X^2^ = 4.30, df = 1, *p* = 0.038). The performances of the two groups of participants varied according to the species (X^2^ = 58.55, df = 2, *p* < 0. 001). Post-hoc tests revealed that participants recognized humans’ facial expressions better than dogs’ (z = 6.75, *p* < 0.001) and cats’ facial expressions (z = 7.48, *p* < 0.001). No significant differences between dogs and cats were found (*p* > 0.05). The type of facial expression of all species influenced the accuracy in both groups of participants (X^2^ = 38.68, df = 4, *p* < 0.001). Anger was recognized more accurately than joy (z = −5.91, *p* < 0.001), neutral (z = −2.73, *p* = 0.049), fear (z = −3.30, *p* = 0.008), and sadness (z = −4.32, *p* < 0.001). Joy was recognized less accurately than neutral (z = 3.68, *p* = 0.002) and fear (z = 3.11, *p* = 0.016).

#### 3.1.2. End Point at (t3)

The model GLMER_binomial_ Accuracy ~ Group + Species + FacialExpression + (1|Individuals) showed that during the third session (t3) **species** and **type of facial expression** influenced the identification of facial expressions. The results were influenced by species (X^2^ = 49.30, df = 2, *p* < 0.001) for both groups and the post-hoc test showed that human facial expressions were identified more accurately than canine (z = 6.20, *p* < 0.001) and feline facial expressions (z = 7.00, *p* < 0.001). For both groups, results were influenced by facial expression (X^2^ = 50.54, df = 4, *p* < 0.001), post-hoc tests showed that, across all species, anger was identified more accurately than joy (z = −5.41, *p* < 0.001), fear (z = −3.27, *p* = 0.009), and sadness (z = −5.18, *p* < 0.001). The neutral facial expression was recognized more accurately than joy (z = 4.99, *p* < 0.001) and sadness (z = −4.75, *p* < 0.001). At (t3) the group (LUSI/control) did not have any significant effect on the results (*p* < 0.05).

### 3.2. Group Dynamic: Exploring the Evolution of the Two Groups with Time

#### 3.2.1. LUSI Group

**Figure 2:**
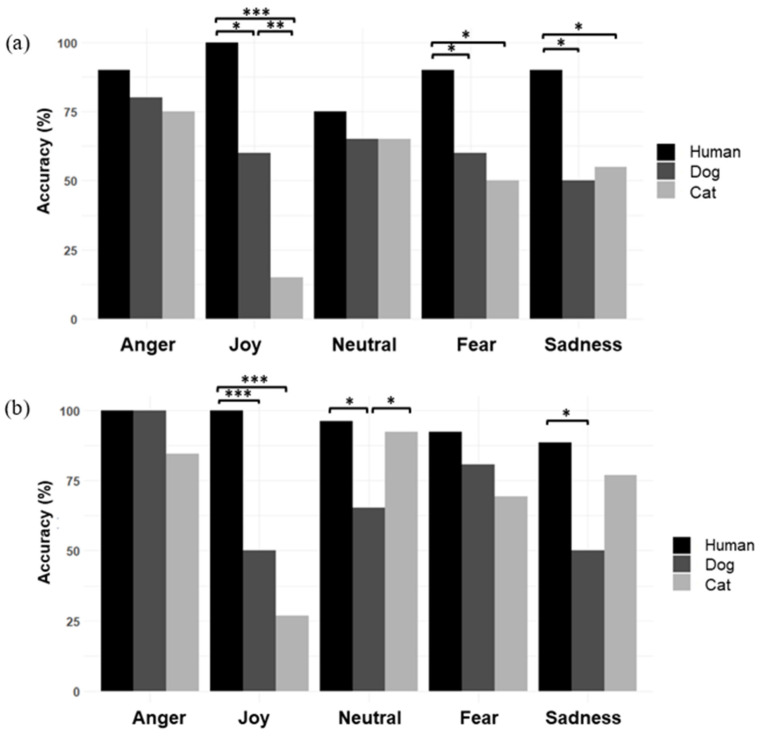
(**a**) Accuracy (%) of the LUSI group for the three species according to facial expression at (t1). (**b**) Accuracy (%) of the control group for the three species according to facial expression at (t1). (McNemar’s Chi-squared test; * *p* < 0.05, ** *p* < 0.01, *** *p* < 0.001).

The GLMER_binomial_ Accuracy ~ Session + FacialExpression+ Species + (1|Individuals) revealed an effect of **session** (X^2^ = 10.90, df = 2, *p* = 0.004), **species** (X^2^ = 10.90, df = 2, *p* = 0.004), and **type of facial expression** (X^2^ = 42.38, df = 4, *p* < 0.001) on the accuracy scores of the LUSI group. Post-hoc tests showed that participants’ accuracy scores were higher at sessions (t2) and (t3) than at session (t1) (t1–t2: z = 3.02, *p* = 0.007; t1–t3: z = 2.58, *p* = 0.027), that participants identified human facial expressions better than feline (z = 9.60, *p* < 0.001) or canine facial expressions (z = 7.22, *p* < 0.001). Dogs’ facial expressions were identified more accurately than cats’ facial expressions (z = 3.80, *p* = 0.0003). Post-hoc tests on facial expressions data showed that, all species combined, anger was identified more accurately than joy (z = −5.41, *p* < 0.001), fear (z = −3.79, *p* = 0.001), and sadness (z = −4.98, *p* < 0.001). Neutral facial expressions were identified more accurately than joy (z = 4.18, *p* = 0.0002) or sadness (z = −3.70, *p* = 0.002). Identification of anger or neutral expressions was not influenced significantly by species (all *p* > 0.05) (e.g., to illustrate, see Figure 2a at (t1)).

Participants identified joy significantly more accurately on human faces than on canine (X^2^ = 6.12, df = 1, *p* = 0.013) or feline faces (X^2^ = 15.06, df = 1, *p* < 0.001) and better on dogs’ than on cats’ faces (X^2^ = 7.11, df = 1, *p* = 0.007). Identification of facial expressions of fear differed according to species, with better identification on human faces than on feline or canine faces (human-cat: X^2^ = 6.12, df = 1, *p* = 0.013; human-dog: X^2^ = 4.17, df = 1, *p* = 0.04), but no significant difference between dog and cat faces (*p* > 0.05).

Finally, differences in the identification of sadness were observed, as it was more accurate for human than for dog and cat expressions of sadness (human-cat: X^2^ = 4, df = 1, *p* = 0.04, human-dog X^2^ = 4.90, df = 1, *p* = 0.02), but did not differ significantly between dogs and cats (*p* > 0.05).

#### 3.2.2. Control Group

The GLMER_binomial_: Accuracy ~ Session + FacialExpression + Species + (1|Individuals) model showed an effect of species and facial expression on control participants’ accuracy, but no effect of session. Analysis of the species effect (X^2^ = 80.28, df = 2, *p* < 0.001) indicated that human facial expressions were identified better than feline (z = 8.88, *p* < 0.001) and canine facial expressions (z = 8.4, *p* < 0.001), with no significant differences between cats and dogs (*p* > 0.05). Post-hoc tests evaluating the effect of facial expression (X^2^ = 98.90, df = 4, *p* < 0.001) indicated that, all species combined, anger was identified more accurately than joy (z = −8.00, *p* < 0.001), neutral (z = −3.17, 0.013), fear (z = −4.39, *p* < 0.001), or sadness (z = −7.11, *p* < 0.001); joy was identified less accurately than neutral (z = 6.48, *p* < 0.001) or fear (z = 5.22, *p* < 0.001); sadness was identified less accurately than neutral (z = −5.23, *p* < 0.001) or fear (z = −3.89, *p* < 0.001) expressions.

Species did not influence significantly identification of anger and fear (all *p* > 0.05) (e.g., to illustrate, see Figure 2b at (t1). Identification of joy differed significantly between the three species: Human expressions of joy were recognized more accurately than canine (X^2^ = 11.08, df = 1, *p* < 0.001) or feline joy (X^2^ = 17.05, df = 1, *p* < 0.001). Identification of neutral facial expressions differed significantly between species: Human neutral expressions were identified more accurately than dog neutral faces (X^2^ = 6.12, df = 1, *p* = 0.013), and cat neutral expressions better than those of dogs (X^2^ = 4, df = 1, *p* = 0.04). Finally, identification of sadness was influenced by species: Accuracy was higher for human than for dog faces (X^2^ = 5.80, df = 1, *p* = 0.02).

### 3.3. Variations with Time of Identification Skills for the Facial Expressions of Our Test Species of Both Groups

#### 3.3.1. Dog Facial Expressions

- Accuracy of the LUSI group

**Figure 3:**
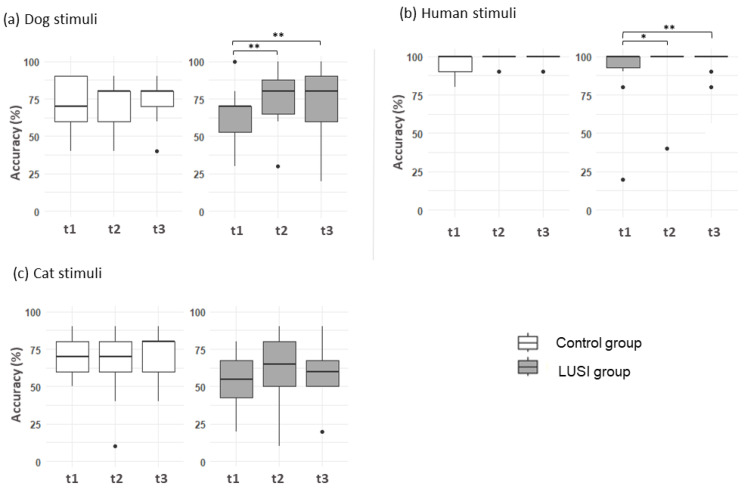
(**a**) Accuracy (%) of identification of dog photographs for each session of control (white) and LUSI (grey) participants. (Accuracy ~ Age + Sex + DogBreed + SCQ + Session + FacialExpression + NbSpecies + (1|Individuals)) (**b**) Accuracy (%) for human photographs according to session and group (GLMER binomial: Accuracy ~ Age + Sex + SCQ + Session + FacialExpression + NbSpecies + (1|Individuals)) (**c**) Accuracy (%) for cat photographs by the control and LUSI groups. (GLMER binomial, Accuracy ~ Age + Sex + SCQ + Session + FacialExpression + NbSpecies + (1|Individuals)). (* *p* < 0.05; ** *p* < 0.01).

The GLMER_binomial_: Accuracy ~ Age + Sex + DogBreed + SCQ + Session + FacialExpression + NbSpecies + (1|Individuals) model revealed an effect of session on accuracy of LUSI participants (χ^2^ = 13.51, df = 2 *p* = 0.001) (Figure 3a). Accuracy increased significantly from (t1) to (t2) (z = 3.39, *p* = 0.002) and from (t1) to (t3) (z = 3.38, *p* = 0.005). The model revealed an effect of age on accuracy: Younger participants more accurately recognized dog facial expressions (χ^2^ = 12.73, *p* = 0.0003). Additionally, accuracy increased with number of different animal species in participant households (χ^2^ = 14.38, *p* = 0.0001). Facial expression also influenced LUSI participants’ performances (χ^2^ = 21.58, *p* = 0.0002): Fear and sadness were identified less accurately than anger (respectively, z = −3.00, *p* = 0.02 and z = −4.42, *p* < 0.001) and sadness was less successfully identified than neutral (z = −2.86, *p* = 0.03). SCQ score, breed of the dog presented, and participants’ sex did not have any significant influence (all, *p* > 0.05).

- Types of errors made by LUSI participants

Chi^2^ tests did not reveal any significant differences concerning the distribution of errors during session (t1) (*p* > 0.05). On the other hand, at (t3), participants confused dogs’ neutral and sadness facial expressions more often (X^2^ = 54.90, df = 18, *p* < 0.001).

- Accuracy of the control group

The GLMER_binomial_ Accuracy ~ Age + Sex + DogBreed + SCQ + Session + FacialExpression + NbSpecies + (1|Individuals) model revealed an effect of sex on accuracy of dogs’ facial expressions, as boys’ scores were lower than those of girls in the control group (X^2^ = 5.55, df = 1, *p* = 0.02). Identification accuracy of the different dogs’ facial expression varied (X^2^ = 36.62, df = 4, *p* < 0.001): Anger was identified more accurately than joy (z = −3.75, *p* = 0.001), neutral (z = −3.16, *p* = 0.01), fear (z = −3.08, *p* = 0.01), and sadness (z = −4.60, *p* < 0.001). Dogs’ sadness was better identified than neutral (z = −3.98, *p* < 0.001) and fear (z = −4.14, *p* < 0.001). The performances of the control participants, contrary to those of the LUSI participants, did not differ significantly between sessions (*p* < 0.05) (Figure 3a). We evidenced no effects of age, number of species in the household, or SCQ score (all tests, *p* > 0.05).

- Types of errors made by the control participants

The most common errors made by adolescents in the control group during session (t1) included frequent confusion of neutral and sadness, as well as fear and sadness (X^2^ = 41.87, df = 18, *p* = 0.001). During session (t3), participants still confused dogs’ expressions of fear and sadness, and neutral and sadness (X^2^ = 61.70, df = 18, *p* < 0.001).

#### 3.3.2. Human Facial Expressions

- Accuracy of the LUSI Group

The GLMER_binomial_ Accuracy ~ Age + Sex + SCQ +Session + FacialExpression + NbSpecies + (1|Individuals) Model showed that session had an effect on LUSI participants’ accuracy (χ^2^ = 8.80, df = 2, *p* = 0.01) (Figure 3b), as their accuracy increased significantly between (t1) and (t2) (z = 2.39, *p* = 0.04) and between (t1) and (t3) (z = 2.93, *p* = 0.008). Additionally, the more participants had different animal species at home, the higher their accuracy (χ^2^ = 11.7, *p* < 0.001). Age, gender, type of facial expression, SCQ score did not have any effect (all tests, *p* > 0.05).

- Type of errors made by the LUSI participants

The distribution of errors did not differ significantly between sessions (t1) and (t3) (all *p* > 0.05).

- Accuracy of the control participants

The GLMER_binomial_ Accuracy ~ Age + Sex + SCQ + Session + FacialExpression + NbSpecies + (1|Individuals) model revealed a significant effect of sex on accuracy, as the boys’ scores were lower than those of the girls (X^2^ = 3.54, df = 1, *p* = 0.059). Other factors, such as age, SCQ score, session, type of facial expression, and number of species present in the household did not have any significant effect (all, *p* > 0.05) (Figure 3b).

- Type of errors made by the control participants

Concerning the distribution of errors identifying human facial expressions, a significant difference was found, as participants confused fear and sadness more in session (t1) (X^2^ = 25.16, df = 9, *p* = 0.003) than during session (t3), when no significant differences were found (all tests, *p* > 0.05).

#### 3.3.3. Cat Facial Expressions

- Accuracy of the LUSI participants

The GLMER_binomial_: Accuracy ~ Age + Sex + SCQ + Session + FacialExpression + NbSpecies + (1|Individuals) model revealed that the lower the LUSI participants’ SCQ score, the higher their accuracy (X^2^ = 9.09, df = 1, *p* = 0.003) (Figure 3c). The more participants had different animal species at home, the more successful they were in this task (X^2^ = 14.70, df = 1, *p* = 0.0001). Facial expression influenced participants’ accuracy (X^2^ = 52.32, df = 4, *p* < 0.001), as joy and sadness were identified less accurately than anger (respectively, z = −6.19, *p* < 0.001; z = −3.18, *p* = 0.013); neutral, fear, and sadness were identified more accurately than joy (respectively, z = 6.32, *p* < 0.001; z = 4.20, *p* < 0.001; z = 3.71, *p* = 0.002); and fear and sadness were identified more accurately than neutral (respectively, z = −2.86, *p* = 0.03; z = −3.38, *p* = 0.006). No effects of session, age, or gender were observed (all tests, *p* > 0.05).

- Types of error made by the LUSI participants

The distribution of errors did not differ significantly between sessions (t1) and (t3), (*p* > 0.05).

- Accuracy of the control participants

The GLMER_binomial_ Accuracy ~ Age + Sex + SCQ + Session + FacialExpression + NbSpecies + (1|Individuals) model showed that type of facial expression influenced control participants’ accuracy (X^2^ = 77.46, *p* < 0.001) (Figure 3c), as joy and sadness were identified less accurately than anger (respectively, z = −7.19, *p* < 0.001; −3.10, *p* = 0.01); neutral, fearful, and sadness were identified better than joy (respectively, z = 7.25, *p* < 0.001; z = 5.93, *p* < 0.001; z = 5.24, *p* < 0.001); and sadness was identified less accurately than neutral (respectively, z = −3.30, *p* = 0.008). Session, age, SCQ, number of different species in the household, or sex did not have any significant effect (all, *p* > 0.05).

- Types of error made by the control participants

The distribution of errors during session (t1) showed that participants more often confused joy and neutral, as well as fear and sadness (X^2^ = 40.67, df = 18, *p* = 0.001), but during session (t3), these pairs of expressions were no longer confused (all tests, *p* > 0.05).

## 4. Discussion

The present study investigated the effects of exposure to a service dog on facial expression identification by a group of schooled adolescents following a special education program (LUSI group) including a service dog. We hypothesized that: (1) the accuracy to identify facial expressions of the LUSI adolescents exposed to a service dog would improve over time and, (2) the variability of confusion between facial expressions would decrease over time. While identification of facial expressions of all test species by the LUSI adolescents was lower than that of the control group during the first session (t1), no significant differences were observed during the last session (t3). This suggests that the two groups did not evolve in the same way during the test year. Indeed, while identification of canine and human facial expressions by LUSI participants (i.e., with service dog present) improved with time, no significant improvement could be evidenced for the control participants (i.e., without service dog). Interestingly, improvement of facial expression identification did not extend to all species. Identification of cats’ facial expressions by the participants of the two groups did not improve with time. Another key finding was the impact of the diversity of species in an adolescent’s household, as well as the type of facial expression, on the identification accuracy of both canine and human facial expressions, and of cats’ facial expressions by the LUSI participants. While adolescents from the control group confused particular pairs of facial expression at both test periods, the LUSI participants showed high variability of types of confusion at (t1). However, 11 to 14 months after the integration of the service dog (i.e., t3) they made a specific confusion similar to that made by the control group (neutral-sadness), for dogs’ facial expressions.

### 4.1. Evolution of Facial Expression Identification

We showed that the ability of LUSI participants, but that not of control participants, to identify both dog and human facial expressions improved from the second session onwards. To our knowledge, a longitudinal study on facial expression identification has yet been carried out on a population with atypical development. Our results are comparable to those of a longitudinal study showing that identification of human facial expression by young children (i.e., 5–7 years old) with typical development improved after participating in dog-assisted intervention sessions and their response latency decreased significantly (Stetina et al., 2011). Those authors suggested that a generalization process from human-dog interactions to human-human interactions had occurred, consistent with our conclusions, since we observed an improvement of identification skills that was not limited to dogs, but that extended to humans. Such generalization processes could be explained by the fact that the service dog within the LUSI group could act as a social catalyst (McNicholas & Collis, 2000; Dollion & Grandgeorge, 2022). As a result, this “social catalyst effect” could lead to a cascade of events: more human-human interactions, and therefore more opportunities to experience and develop social skills, such as facial expressions processing. The service dog’s presence and its spontaneous behaviour are said to redirect social attention, eliciting more social interactions between humans. Due to a long history of coexistence with humans, dogs display behaviours considered to be facilitators of pseudo-social (human-dog) and social (human-human) interactions (e.g., curiosity, playful nature, easy to encounter; Dicé et al., 2017). The results of this reorientation of social attention were relayed by Dollion et al. (2022), who reported that the visual exploration strategies during human facial expression identification by children with ASD who were recipients of a service dog was better than that of children with ASD without a service dog (Dollion et al., 2022). Frequent interactions of young people with ASD with a service dog could promote the development of more efficient visual exploration strategies connected to processing human facial expressions. An alternative explanation would be that the ability to recognize dogs’ and humans’ facial expressions could be linked to humans’ ability to identify their own facial expressions. Indeed, while interactions with humans can be complicated and unattractive for some adolescents (particularly with ASD), dogs can be attractive and motivating interaction partners. Interactions with a dog can arouse emotions in both adolescents and service dogs. This, in turn, encourages them to observe, self-examine and become more sensitive to their own feelings and the service dog’s behaviour. This can sometimes induce them to express their feelings, whether by verbalizing about the service dog to other classmates or to the teacher, or by speaking directly to the service dog, thus enabling them to become more aware of their emotions. This hypothesis is supported by the aforementioned child study, which showed that animal-assisted interventions help children with typical development to recognize and understand their own emotions better, as well as those of their companions and animals (Dicé et al., 2017). However, younger children (between 3.5 and 6 years old) may have difficulty to understand dogs’ facial expressions correctly, even after receiving training in canine emotions as part of an educational intervention (Morrongiello et al., 2013). Another possible explanation is that processing human and canine facial expressions involves similar brain processes and/or visual exploration strategies (Dollion et al., 2022). For example, the visual strategies of typically developing individuals for processing animal and human faces are similar (i.e., so-called “triangular” exploration of eyes and mouth; Grandgeorge et al., 2016; Valiyamattam et al., 2020). Additionally, similar brain areas are involved in processing human and animal faces, and activation of these areas does not appear to be altered in people with ASD when processing animal faces (Blonder et al., 2004; Whyte et al., 2016).

Identification of facial expressions improved for both dog and human faces, but this improvement did not extend to cat faces, and this even though cats are a species with discreet emotional expressions (Scott & Florkiewicz, 2023). Humans probably find identification of these signals more challenging. Furthermore, the present result could be explained by the fact that cats have a facial morphology and emotional expression patterns different to those of dogs, as well as different interaction styles. Consequently, daily exposure to dogs may not be beneficial for feline facial expression identification, or such improvements may take longer to emerge. Furthermore, here, we tested only one sensory modality (e.g., vision) for facial expression identification and one body area (i.e., pictures incorporating just the head; no body posture, tail movements/position, hair ruffling and so on). A study of adults showed that signals from cats are better understood when visual and vocal cues are combined (De Mouzon et al., 2024).

Participants who did not benefit from daily exposure to the service dog showed no such improvement. These results agree with a study conducted on typically developing children aged 6–7 years in school (Hergovich et al., 2002). Indeed, this study compared the effects of a dog’s presence in the classroom on empathy towards animals and social intelligence of children among pupils in two different classrooms: one including a dog for three months, the other without a dog. The authors did not find any improvement in recognition of human facial expressions (e.g., happiness, surprise, sadness) by either the experimental or the control participants. So, to draw a parallel with our results, short-term exposure to a dog does not induce improvement in recognition of human facial expressions in typically developing children. It is important to note that a “plateau phenomenon” cannot be excluded in this group concerning the human stimuli. Indeed, not until they are 10–11 years old does a child’s ability to interpret others’ emotions reach a level comparable to that of adults (Tonks et al., 2007). Thus, our task may have been too simple for the control group, and they may have already excelled in the first session, leaving little opportunity for improvement. However, control adolescents confused fear and sadness during the first session more than at (t3). This result suggests that, for the control group, no maturation, or age-related evolution effect on emotion identification skills was present over the tested period. Unlike our LUSI participants, our control participants’ identification of the dog stimuli showed no improvement, and they continued to demonstrate the same types of confusion. As these adolescents did not have the opportunity to interact with the service dog, they were thus not able to familiarize and learn from interactions with it, and in turn their skills did not evolve.

### 4.2. Identification Skills for the Facial Expressions of Three Different Species of Adolescents With or Without Atypical Development

Our study showed that both groups of adolescents recognized human facial expressions better than canine and feline facial expressions. Humans may encounter obstacles to understand other species’ facial signals, as emotional signals have evolved to facilitate intra-species rather than inter-species communication (Hawkins et al., 2021). Moreover, humans’ attraction to faces appears very early during development and specialization in the processing of human faces appears between 6 and 9 months of age (Pascalis et al., 2002). Additionally, recognition of happiness, sadness, and anger develops early in life and accuracy of their recognition remains consistently high from 6 to 16 years old (Lawrence et al., 2015).

Our study showed that anger expressed by our three tested species was well-identified by both groups of adolescents. Certain canine facial expressions, such as anger and joy, are well categorized by children, regardless of their experience (Amici et al., 2019). Lakestani et al. (2014) showed that children (aged 6, 8, and 10 years) were better at interpreting dogs’ emotions in defensive-aggressive conditions (correct answers: 92%) but poorer in fearful conditions (41%), friendly conditions being at an intermediate level (57%). Both our groups confused more neutral and sadness on dog faces (and fear and sadness on cat faces by the control participants only) but not on human faces (Lakestani et al., 2014). The presence of ASD traits can affect the ability to recognize human facial expressions of sadness and anger, resulting in a tendency to categorize both expressions as sadness (Green & Guo, 2018). Lastly, anger expressed by all three species is an emotional particularly recognizable facial expression, as it involves easily identifiable features (i.e., teeth displayed by animals, mouth opened at various degrees, pronounced frowning). In contrast, dogs’ and cats’ positive facial expressions could be more difficult to identify by our human eyes, as key human features characteristic of joy (Duchenne smile) are not displayed by these species (Girard et al., 2019).

### 4.3. Factors Influencing Facial Expression Identification

Our study showed that exposure of adolescents to animals outside the school environment influenced their ability to identify facial expressions. Indeed, our results showed that adolescents who had a wide variety of different species at home succeeded the facial expression identification tests better, regardless of the species. A first explanation would be that being in contact with a greater variety of species at home could facilitate a greater variety of experiences of facial expression processing and recognition, leading to an overall improvement in the ability to identify facial expressions. Amici et al. (2019) reported that the ability to categorize dogs’ facial expressions is acquired mainly through experience (Amici et al., 2019). However, other authors contradict these effects of experience (Demirbas et al., 2016; Schirmer et al., 2013). Another identified factor of variation was gender, as girls in the control group identified dogs’ facial expressions better than boys did. This result echoes the report that women are more sensitive to affective information than males and better identify human and canine expressions (Schirmer et al., 2013). Moreover, in classroom settings with dogs present, 6- to 7-year-old girls generally pay attention to dogs longer than do boys of the same age (Kotrschal & Ortbauer, 2003).

### 4.4. Limits

One of the main limitations to the present study is the size of our sample, due to the small number of adolescents enrolled in the LUSI program (i.e., only a small number of students in each college), and to the longitudinal design, which added additional challenges; although none of the participants stopped participation during the study. Second, although participants’ SCQ scores and Dunn profiles were homogeneous in each group, disorders of the LUSI group were heterogeneous. Furthermore, the control and LUSI groups differed on several parameters, suggesting the need for a future study with a control group consisting of a LUSI class without a service dog. Another limitation of this study lies in the heterogeneity of diagnoses within our group of LUSI adolescents, which likely introduced some variability into the results obtained. However, this heterogeneity reflects the inherent structure of the national LUSI program in France, designed to support youth with academic difficulties regardless of their specific diagnoses. While these diagnoses are diverse, they share common features related to cognitive difficulties. This heterogeneity in diagnoses may have influenced the conditions under which the facial expressions of the service dog were observed, especially since there is no documentation regarding the actual possibilities of observing these expressions under different conditions. It is possible that the nature of direct “face-to-face” interactions varied depending on the adolescents’ diagnoses, which may have influenced their perception of the service dog’s facial expressions. It would have been interesting to adopt a more individualized approach, such as a case study, in order to explore the incidence of children’s specific profiles in greater depth. A limitation of the SCQ measure used in this study is that the version employed was designed for children aged 3 to 10 years old, whereas our adolescent population was aged 11 to 15 years old. Maybe our study should have included more photographs of facial expressions; however, pre-tests prompted us to limit our choice to a maximum of 30 photographs, due to the high fatigability of LUSI adolescents. In addition, a plateau phenomenon seemed to be observed for the identification of human facial expressions, meaning that the task may have been too simple for the control participants. Integrating photographs with more subtle facial expressions could be of interest in the future to complexify the task. Similarly, the presentation format of the stimuli (e.g., photographs of faces) can be questioned. Stimuli depicting dynamic characters and animals could be considered, and/or representing the whole body of the animal and depicting its posture. Such types of stimuli would be more realistic and could constitute more complex and richer cues to process. The types of interactions adolescents had with the dog were not collected in this study; investigating if the types of interactions and relationship adolescents establish have any influence on benefits should be considered in future studies. Finally, neither the teachers’ role in the interactions and dynamics within the classroom, nor their role in the interactions with the service dog have been considered here and could be, in future studies, explored to deeper understand the service dog role.

## 5. Conclusions

Although well-documented in the home environment, the benefits of animals in the school environment have yet to be fully explored. A recent review of the literature on the impact of therapy dogs on students reported improvement of their mood, social adeptness, effective communication skills, self-assurance, and dynamics within the teacher–student relationship (Wintermantel et al., 2024). However, it must be borne in mind that these studies present considerable variability in terms of type of intervention, data collection, number of participants and duration of exposure to the animal (Brelsford et al., 2017). Future studies should therefore focus on standardizing practices and observation methods, so as to produce more repeatable studies. It would of interest for future studies, instead of focusing solely on the face scale and using static stimuli, to use full-body stimuli (e.g., including posture and even behavioural sequences), and to consider incorporating dynamic stimuli to further explore if the use of more naturalistic stimuli has an influence on the reading of emotions.

To conclude, our study is the first to demonstrate the long-term benefits of the presence of a service dog at school on facial expression identification skills of adolescents with various cognitive disorders. Regular exposure to a service dog in the school context resulted in an improvement of both canine and human facial expression identification skills in these adolescents after 5–8 months of exposure. This improvement was not observed in the control group, who had no exposure to the service dog. Furthermore, while the identification of facial expressions on all tested species by the LUSI adolescents was lower than in the control group during the first session (t1), no significant difference was observed during the last session (t3), suggesting a convergence between the LUSI group’s performances toward that of the control group. While consistent with the existing literature on the benefits of animals for young people, the scientific literature has yet to explore further their effects in the school environment (Wintermantel et al., 2024). Lastly, these results highlight the potential applications of such interventions, particularly for addressing children’s and adolescents’ specific emotional and psychological difficulties. Supplementing our observations with data acquired using an eye-tracker while processing human and canine faces could be of interest to shed new light on the mechanisms involved in the improvement of facial expression identification skills observed in the present study.

## Figures and Tables

**Table 1:** General characteristics of the adolescents included in the study: (**a**) LUSI group (a SCQ score > 15 was considered as indicative of probable ASD); (**b**) Control group. ADD: Attention deficit disorder, ASD: Autism Spectrum Disorders, DYS: Dyslexic disorder, IDD: Intellectual and Developmental Disorders. (t1): beginning of the study. ^a^ According to their parent.

**(a) Adolescents with Service Dog—LUSI Group**								
**Participants’ Number**	**Age (yo) at (t1)**	**Sex**	**Vision’s Problem** **(Glasses)**	**Diagnosis**	**Number of Species at Home**	**Strong Bond with Their Pet ^a^**	**SCQ** **Score**	**Dunn** **Score**
**1**	12	M	YES	Left hemiparesis, ADD	1	NO	11	130
**2**	14	F	NO	DYS Disorders, mild IDD	2	YES	8	158
**3**	15	F	NO	ADD	2	YES	13	141
**4**	12	M	YES	Epilepsy	1	NO	2	160
**5**	11	F	YES	ASD	1	NO	18	166
**6**	15	M	YES	Cutis laxa	3	NO	25	180
**7**	12	M	NO	DYS Disorders	1	YES	22	161
**8**	15	M	NO	ASD	0	NA	23	126
**9**	14	M	NO	ASD	4	YES	14	169
**10**	13	F	YES	DYS disorders, genetic disease	1	YES	14	170
**(b) Adolescents Without Service Dog—Control Group**								
**Participants’ Number**	**Age (yo) at (t1)**	**Sex**	**Vision’s Correction (Glasses)**	**Diagnosis**	**Number of Species at Home**	**Strong Bond with Their Pet ^a^**	**SCQ Score**	**Dunn Score**
**1**	12	M	YES	None	1	YES	1	119
**2**	13	F	NO	None	1	NO	8	151
**3**	12	M	NO	None	2	YES	1	-
**4**	11	F	NO	None	1	YES	2	177
**5**	13	M	NO	None	2	YES	2	170
**6**	13	M	NO	None	2	YES	3	153
**7**	13	F	NO	None	3	YES	3	150
**8**	12	M	NO	None	1	NO	5	142
**9**	13	F	NO	None	1	NA	3	-
**10**	14	F	NO	None	0	NA	5	164
**11**	12	M	NO	None	4	YES	6	177
**12**	13	M	NO	None	1	NO	1	182
**13**	12	F	YES	None	4	NO	5	168

## Data Availability

Data will be available on authors request.
